# Insights in the Role of Lipids, Oxidative Stress and Inflammation in Rheumatoid Arthritis Unveiled by New Trends in Lipidomic Investigations

**DOI:** 10.3390/antiox10010045

**Published:** 2021-01-02

**Authors:** Helena Beatriz Ferreira, Tânia Melo, Artur Paiva, Maria do Rosário Domingues

**Affiliations:** 1 Mass Spectrometry Center & QOPNA/LAQV-REQUIMTE, Department of Chemistry, Campus Universitário de Santiago, University of Aveiro, 3810-193 Aveiro, Portugal; helenabeatrizferreira@ua.pt; 2 CESAM, Centre for Environmental and Marine Studies, Department of Chemistry, Campus Universitário de Santiago, University of Aveiro, 3810-193 Aveiro, Portugal; 3 Unidade de Gestão Operacional em Citometria, Centro Hospitalar e Universitário de Coimbra (CHUC), 3004-561 Coimbra, Portugal; artur.paiva@chuc.min-saude.pt; 4 Coimbra Institute for Clinical and Biomedical Research (iCBR), Faculty of Medicine, University of Coimbra, 3000-370 Coimbra, Portugal; 5 Instituto Politécnico de Coimbra, ESTESC-Coimbra Health School, Ciências Biomédicas Laboratoriais, 3046-854 Coimbra, Portugal

**Keywords:** rheumatoid arthritis, lipidomics, mass spectrometry, biomarkers, lipid peroxidation

## Abstract

Rheumatoid arthritis (RA) is a highly debilitating chronic inflammatory autoimmune disease most prevalent in women. The true etiology of this disease is complex, multifactorial, and is yet to be completely elucidated. However, oxidative stress and lipid peroxidation are associated with the development and pathogenesis of RA. In this case, oxidative damage biomarkers have been found to be significantly higher in RA patients, associated with the oxidation of biomolecules and the stimulation of inflammatory responses. Lipid peroxidation is one of the major consequences of oxidative stress, with the formation of deleterious lipid hydroperoxides and electrophilic reactive lipid species. Additionally, changes in the lipoprotein profile seem to be common in RA, contributing to cardiovascular diseases and a chronic inflammatory environment. Nevertheless, changes in the lipid profile at a molecular level in RA are still poorly understood. Therefore, the goal of this review was to gather all the information regarding lipid alterations in RA analyzed by mass spectrometry. Studies on the variation of lipid profile in RA using lipidomics showed that fatty acid and phospholipid metabolisms, especially in phosphatidylcholine and phosphatidylethanolamine, are affected in this disease. These promising results could lead to the discovery of new diagnostic lipid biomarkers for early diagnosis of RA and targets for personalized medicine.

## 1. Rheumatoid Arthritis

Rheumatoid arthritis (RA) is a chronic inflammatory autoimmune disease (AID) affecting almost 1% of the population worldwide and is most prevalent in women [1]. In RA, chronic inflammation of the synovium membrane is the crucial pathologic feature, leading to progressive and irreversible joint destruction, deformity, and disability, if left untreated [2]. Increased autoantibody production is another hallmark of RA, in particular, the rheumatoid factor (RF) and the anticitrullinated protein antibodies (ACPA) [3]. Both RF and ACPA have predictive, diagnostic, and prognostic roles, since it is possible to detect these autoantibodies prior to RA onset and their presence positively correlates with disease severity and joint destruction [4,5]. However, only about 70% of RA patients produce RF or ACPA [6], thus revealing that the diagnosis of RA cannot be based solely on these biomarkers.

Besides the presence of autoantibodies, the etiology of RA is complex, multifactorial, and is yet to be completely elucidated. The main physiological characteristics of RA (joint inflammation and cartilage destruction) are the result of the infiltration of immune cells into the synovial joint lining and the associated complex network of cytokines [3,7,8]. Impaired adaptive immune responses are considered keys in RA pathogenesis. In brief, activated T helper cells release several pro-inflammatory cytokines into the synovial membrane and fluid [9]. Another set of pro-inflammatory cytokines is secreted by recently activated macrophages contributing to a self-amplifying pro-inflammatory loop. This induces the proliferation and differentiation of B cells, resulting in the production of RF and ACPA autoantibodies [3]. Fibroblasts from the synovial membrane produce matrix metalloproteinases that will destroy the cartilage and activate osteoclasts, promoting bone resorption. The release of such pro-inflammatory mediators induces protein modifications that enhance the immune response and chronic inflammation [10,11].

Additional pivotal cells for RA pathogenesis are monocytes and neutrophils. It has been suggested that an increased turnover of monocytes, migrating from the bone marrow into the inflamed synovia, occurs in RA [12]. The process of recruiting neutrophils into the synovium is a key feature of inflammatory responses in RA, and it is propelled, once more, by an intricate network of cytokines [3]. The aforementioned pro-inflammatory cytokines released by macrophages and T cells serve as a primer of the infiltrated neutrophils. Once primed, neutrophils will foster joint destruction by inducing synthesis of proteins that can upregulate and extend neutrophil ability to secrete a large quantity of cytokines and chemokines [13,14]. Furthermore, neutrophils contribute to cartilage destruction in the synovial fluid, damage surrounding tissues, induce oxidative stress conditions due to the release of reactive oxygen species (ROS), increasing the inflammation status, and may be a source of ACPA contributing for the impaired immune response in RA [15,16,17].

Given the complex character of RA, it is believed that genetic and environmental factors also take part in the pathogenesis of this disease. RA susceptibility is strongly associated with major histocompatibility complex genes, and the genetic factors have a straight connection with the environmental factors [18]. The production of ACPA may be triggered by silica exposure, microbiota, infections, epigenetic modifications and environmental risk factors, specially cigarette smoking [19]. The microbiota of RA patients differs from that of the general population and is associated with the inflammatory conditions of this disease, while viral and bacterial infections contribute for the development of the autoimmune response in RA [20]. Additionally, RA has an increased heritability that is estimated to be 50% for ACPA-seronegative and 70% for ACPA-seropositive [21,22]. The expression of ACPA in RA patients is associated with the presence of shared epitope alleles (SE) which, in turn, are associated with disease severity [23]. In genetically predisposed individuals, the environmental factors have a high impact on the risk of developing RA by inducing molecular changes to proteins that trigger a loss of immunological self-tolerance [3]. 

Systemic effects linked to inflammation are responsible for severe physical disability of RA patients, diminishing their life quality. Impaired muscle function may be considered a hallmark of RA and several factors contribute to this process. Pro-inflammatory cytokines, as mentioned before, are highly involved in RA pathogenesis. Cytokines are responsible for low skeletal muscle function in RA patients by reducing muscle fibers contractility due to increased oxidant activity in the system [24]. Cytokines were found to be two times higher in RA patients’ muscle compared with systemic cytokines levels [25]. The chronic inflammation in RA is also involved in skeletal muscle pathology. Inflammation and insulin resistance are correlated with diminished mitochondrial function and content, thus disrupting muscle oxidative metabolic capacity and increasing levels of intramyocellular lipid content [26,27]. RA patients have an increased risk for developing insulin resistance, which is influenced by RF seropositivity, higher disease activity, prednisone use, and visceral and thigh intermuscular adiposity [24,28]. The increased accumulation of toxic lipid mediators and consequent lipotoxicity in skeletal muscles may also stimulate mitochondrial dysfunction, that, together with insulin resistance, contribute to cardiovascular disorders and sarcopenia [29]. Insulin resistance, cardiovascular disorders, and sarcopenia, associated with an increased intramuscular fat compared to healthy subjects, are frequently associated with RA [30].

**Figure 1:**
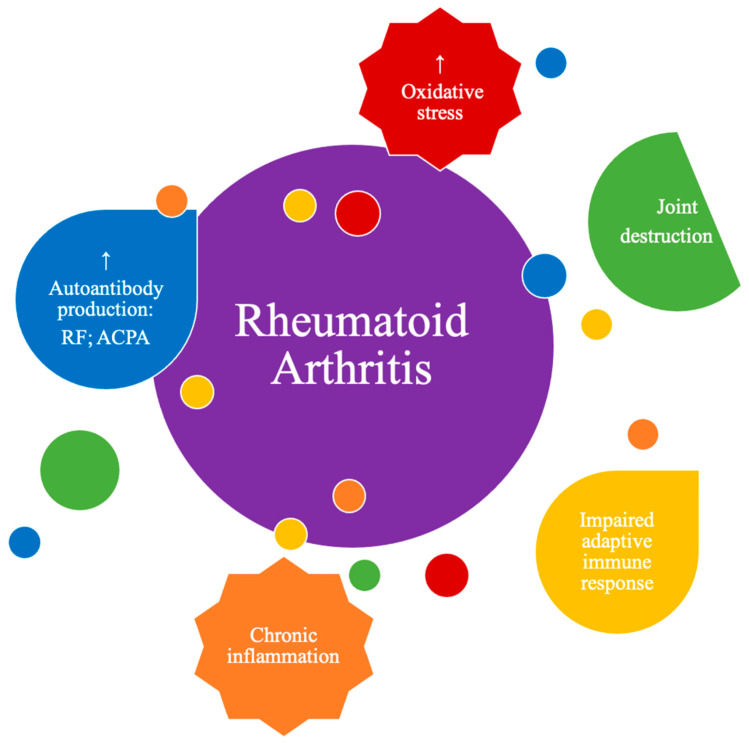
Main physio-pathological alterations that occur in rheumatoid arthritis (RA).

Although the true etiology of this chronic inflammatory, rheumatic and immune-mediated disease remains unknown, several factors are known to contribute to the development and pathogenesis of RA, (Figure 1), namely inflammation exacerbation, oxidative stress and lipid peroxidation, as will be detailed in the following chapters. 

## 2. Oxidative Stress in Rheumatoid Arthritis

RA pathogenesis is associated with increased oxidative stress, decreased antioxidant levels, and impaired antioxidant defenses [20]. Reactive nitrogen species (RNS) and ROS have been implicated in RA pathogenesis, when they exceed physiological concentrations, especially through the damage of lipids of plasmatic membranes, proteins, and nucleic acids [31]. In fact, a fivefold increase in mitochondrial ROS production has been identified in whole blood and monocytes from RA patients, suggesting oxidative stress to be a pathogenic characteristic in RA [32].

The release of cytokines by T cells induces oxidative stress, thus antioxidants, such as glutathione, are important to reduce the oxidative status. Vitamin C and vitamin E are important non-enzymatic antioxidants essential to, for instance, immune system functions, repair body tissues and protect cell membranes from oxidative damages. However, evidence suggests that the antioxidant system in RA has been compromised. Serum vitamin C levels [33] and plasma concentration of vitamin E [34] were significantly lower in RA patients, re-enforcing the fact that oxidative stress impairs the antioxidant system in RA. Additionally, low levels of tocopherols, β-carotene, retinols, SH groups and L-γ-glutamyl-L-cysteinylglycine have been found in RA patients along with low activity levels of glutathione reductase and superoxide dismutase [35,36]. However, these findings are not consistent in every study on enzymatic activity in RA patients. Superoxide dismutase, glutathione peroxidase, catalase, glutathione reductase, NADPH oxidase, myeloperoxidase, and arylesterase have been found to be increased, decreased or even without altered activity in different investigations (for further information, please consult the review article [32]). Nitric oxide (NO•) free radical negatively correlates with glutathione, which could be a compensatory effect of intracellular non-enzymatic antioxidative mechanisms to an increased nitrogen dioxide (NO_2_•) production in RA [37]. Also, the suppression of endothelial NO• synthase activity was found to be considered a characteristic of endothelial injury, leading to atherosclerosis, which is common in RA [38]. By these contradictory results, it is possible to assume that the antioxidant systems of RA patients are impaired, requiring more studies to clarify this matter.

The immune response may become abnormal due to T cell exposure to increased oxidative stress by disturbing growth and death stimuli [39]. Furthermore, neutrophils from RA produce higher quantities of neutrophil extracellular traps (NET), ROS, and RNS than healthy people [3]. NETosis, the process of NET formation, is augmented in both circulating and synovial neutrophils of RA and is linked with ACPA seropositivity and inflammatory markers. NETosis contributes to the production of ACPA, which results in pro-inflammatory molecules synthesis [16]. This way, NET may be considered a potential biomarker of RA early diagnosis because the concentration of plasma levels of cell-free nucleosomes shows high sensitivity (91%) and specificity (92%) [40,41].

DNA oxidation products, such as 8-oxo-7-hydro-deoxyguanosine, are produced due to the reaction between hydroxyl radicals and deoxyguanosine and are found in serum and lymphocytes of RA [42,43]. The levels of oxidation of uric acid were higher in RA patients as well [44]. Protein oxidation has also been reported in RA. Oxidative stress leads to an accumulation of advanced oxidation protein products [45]. Protein oxidation markers have been found in higher concentrations in plasma and synovial fluid of RA patients and were correlated with disease progress and development [44,46,47,48,49]. Higher levels of protein carbonyls and 3-chlorotyrosine were detected in synovial fluid, and other types of RA samples, along with increased activity of myeloperoxidase, which catalyzes the reaction between hydrogen peroxide (H_2_O_2_) and chloride anion (Cl-) producing hypochlorous acid (HOCl) that can cause oxidative damage in the host tissue. This way, the increase of myeloperoxidase activity suggests a pronounced oxidative stress environment [44,50]. Hypochlorous acid can react with nitrite (NO^2−^), resulting in nitrate (NO^3+^), which was found to be increased in serum of RA patients [51]. Moreover, myeloperoxidase is known to convert low-density lipoproteins (LDL) into a foam cell forming LDL isoform and promote the formation of dysfunctional high-density lipoproteins (HDL), thus not only contributing for oxidative stress conditions but also for a dysregulated lipid metabolism and an atherogenic environment [52]. Synovial fluid is rich in hyaluronic acid (2–3 mg/mL), a glycosaminoglycan (GAG) which is a linear homopolymer of the disaccharide repeating units of [D-glucuronic acid-beta-(1-3)-N-acetyl-D-glucosamine] linked together with beta-(1-4) glycosidic linkages. Hyaluronic acid, like other GAGs and proteins, is highly reactive with ROS and RNS. The modifications of hyaluronan in synovial fluid are highly informative about the pathophysiological condition of synovial joints [53]. The balance between the superoxide anion radical and NO radical precursors, the intermediates such as H_2_O_2_, and the degradation products like the hydroxyl radical, determines which GAG component (i.e., hyaluronan, heparan sulphate, chondroitin sulphate) of the extracellular matrix is predominantly degraded and may be important in regulating disease processes.

This way, oxidative damage biomarkers have been consistently found to be significantly higher in RA patients than in controls in any type of sample analyzed. Altogether, it is well established that prevails an oxidative stress environment in RA, leading to biomolecules oxidation and stimulating inflammatory responses in this debilitating pathology. Lipids are biomolecules that are very susceptible to the attack of ROS and RNS under oxidative stress conditions. Therefore, lipid peroxidation is one of the major consequences of oxidative stress. Cigarette smoking for instance, which is an environmental factor that triggers RA development, is a source of exogenous lipid peroxidation products, which are capable not only of promoting the depletion of serum antioxidants that normally act as radical scavengers, but also of affecting directly biological macromolecules, by introducing third-party carbonyl groups into a wide array of proteins, such as cytoskeletal proteins, glycolytic enzymes, antioxidant enzymes, and endoplasmic reticulum proteins, and by attacking the free amino-groups of DNA bases [54,55,56]. Lipids, including phospholipid oxidation products, are important in the modulation in an inflammatory response [57]. In addition, oxidative stress and inflammation may also be associated with an increased dysregulation of the lipid metabolism, which is also found in RA. Thus, in the next chapter we will detail the role of lipids in RA.

## 3. Lipids Are Key Players in Rheumatoid Arthritis

Lipid metabolism alterations have been suggested to participate on RA pathogenesis and to contribute to disease severity. Lipid peroxidation is also an important consequence of oxidative stress. When lipids are oxidized, fluidity, and permeability of plasmatic membranes are altered, and membrane-bound enzymes may become dysfunctional [32]. Oxidation of lipids leads to the formation of lipid hydroperoxides, which are quite unstable molecules, and can decompose and lead to the formation of several bioactive aldehydes like 4-hydroxynonenal (HNE), malondialdehyde (MDA), which are electrophilic molecules that can react with proteins, leading to changes in their function [58]. MDA levels have been reported to be significantly higher in RA whole blood, plasma, serum, synovial fluid, erythrocytes and urine [34,39,42,59,60,61,62,63,64]. MDA levels have also been positively correlated with disease activity and levels of ROS [49]. MDA is highly reactive and can spontaneously break, resulting in acetaldehyde. MDA and acetaldehyde can react with each other, leading to the formation of malondialdehyde-acetaldehyde (MAA) protein adducts, which the production was increased in RA synovial tissue [65]. Besides, a correlation was also found between increased levels of anti-MAA antibodies and seropositivity for RF and ACPA, revealing a potential pathogenic role of this biomarker. Additionally, ACPA seropositivity was linked with increased MDA and myeloperoxidase levels in RA synovial fluid [59]. Thiobarbituric acid reactive substances (TBARS), usually used to measure the levels of lipid oxidation and lipid carbonylated species, were found markedly elevated in RA blood as well [37,46,66]. Isoprostanes, formed by enzymatic oxidation of lipids, excretion, and plasmatic concentrations were identified as significantly higher in RA patients than in controls [64,67,68]. Isoprostanes excretion was also positively correlated with diminished HDL protective effect against coronary calcification in patients with RA [67].

The overproduction of eicosanoids, like the ones observed in RA, could be correlated with dietary polyunsaturated fatty acids (PUFA). Eicosanoids directly derive from ω-3 and ω-6 PUFA esterified to phospholipids (PL) of the plasmatic membranes of immune cells and were correlated with PUFA dietary intake [69]. It is well established that arachidonic acid (AA, fatty acid (FA) 20:4) derived metabolites have a pro-inflammatory effect while eicosapentaenoic acid (EPA, FA 20:5) and docosahexaenoic acid (DHA, FA 22:6) derivatives promote an anti-inflammatory environment. Therefore, dietary ω-3 PUFA may have favorable clinical applications in patients with RA [70]. Evidence suggests that there is an inverse correlation between the intake of ω-3 PUFA, and RA prevalence. Populations whose intake of ω-3 PUFA is higher have a lower incidence of RA [71,72]. A similar correlation was found to olive oil consumption, however in this case it is due to the increase of C18:1 consumption, which is less prone to oxidation, than the increased intake of ω-3 PUFA [73,74]. Clinically, ω-3 PUFA supplementation has been shown to reduce morning stiffness, lower tender, swollen, and painful joints, improve global arthritic activity, ameliorate biological parameters of inflammation and reduce drug requirement for disease management (for further information, please see review article [70]). ω-3 PUFA supplementation has beneficial effects reducing the symptoms of RA. However, it cannot be considered a standard treatment of this pathology.

### Dyslipidemia as an Important Contributor to RA Pathogenesis

The increased concentrations of pro-inflammatory cytokines reported throughout the previous chapters are responsible not only for favoring oxidative stress conditions but also for contributing to dyslipidemia in RA patients [45]. Dyslipidemia is prevalent in 55–65% of RA patients and it can be detected in its early stages or even before the diagnosis of clinical RA, a time in which both inflammation and autoimmunity (RF, ACPA) are typically elevated [75,76]. Both active or untreated RA is associated with an unfavorable lipoprotein profile, which is essentially characterized by decreased serum levels of HDL, and variation in levels of LDL and total cholesterol (TC) [77,78]. LDL and TC levels have contrasting results in several studies [79,80,81]. These discrepancies may be due to differences in the study designs such as samples sizes, populations, treatment effects and unconsidered confounders. The reduction of HDL leads to the increase of TC/HDL ratio which was correlated with disease activity and representative of an atherogenic index, thus being a relevant prognostic marker for the risk of cardiovascular diseases [81]. Moreover, a variation was reported in the composition of HDL PL that may be accountable for the decrease in its atheroprotective function, besides explaining the higher cardiovascular risk in RA [64,82].

Singh et al. saw significantly decreased levels of HDL and TC and increased levels of LDL in RA [83]. The reduction of TC levels was less pronounced than the HDL levels, resulting in an increase of both TC/HDL (atherogenic index) and LDL/HDL ratios [83]. Lower levels of TC and HDL were associated with TNF-α and higher levels of disease activity, which could be an explanation for the correlation between disease activity and lipoprotein levels [83]. Cacciapaglia et al. observed significantly lower levels of TC, LDL, triglycerides, and atherogenic index in RA patients with low disease activity when compared with patients with moderate/high disease activity [84]. It should be noted that there is a lipid paradox in RA where the conventional hypercholesterolemia link with cardiovascular diseases is not straight forward. It has been reported that patients with lower levels of TC, LDL, and atherogenic index had an increased risk of cardiovascular diseases than those with higher levels of lipoproteins, suggesting that hypercholesterolemia as a risk factor for cardiovascular diseases may not be applicable in RA [85]. Moreover, growing evidence suggests that specific lipoprotein subfractions are altered as well, which may contribute to the initiation and progression of atherosclerosis in RA patients [86]. All things considered, although TC and LDL levels are inconsistent in several studies, and may resemble those of the healthy population, the lipoprotein sizes, the apolipoprotein cargo of lipoproteins and the lower levels of HDL in RA patients are enhancers of a pro-atherogenic dyslipidemia [78].

Oxidized LDL (ox-LDL) has also been reported in RA. A significant elevation of the ox-LDL fraction has been found in RA patients with and without carotid plaques [87,88], enhancing pro-atherogenic events, inflammatory responses through upregulation of chemokines, adhesion molecules and production of advanced glycation end-products, improving the risk of RA patients to develop cardiovascular complications [45,83,89]. Chronic inflammation also leads to HDL oxidation and reduced apolipoprotein-A-I in patients with active RA [90].

Changes in the lipoprotein profile seem to be a hallmark of RA and lipid peroxidation is also present in this disease. Dyslipidemia may, in fact, be responsible for the increased risk for cardiovascular diseases in RA. Nevertheless, the interaction between oxidative stress, inflammation and changes in the lipid profile, and the functionality of lipoproteins in RA are still poorly understood, and there are still conflicting results between studies revealing the need for more research in this field. Thus, dysregulation of lipid homeostasis and lipid metabolism at a molecular level was also reported, as will be detailed in the next chapter.

## 4. Lipidomic Studies in Rheumatoid Arthritis

From the evidence described in the previous chapter, it is accepted that, in fact, there are changes in the lipid metabolism in this pathology. The correct regulation of the lipid metabolism is vital for the homeostasis of the organism, thus avoiding pathological states. The dysregulation of lipid homeostasis affects several processes of inter and intracellular signaling and regulation of the immune response. Thus, the analysis of lipids and their variation is fundamental for a better understanding of the pathogenesis of several chronic diseases such as RA. Personalized medicine is the utmost tailored approach to disease management. Although the routine substances work for the general population, there are some patients to whom the drug effects are not the desired ones. To overcome this setback, biomarkers are of extreme importance to exclude the undesired effects of the drug treatment. These biomarkers may be autoantibodies, genes, or even biomolecules such as lipids. It should be noted that RA treatment involves corticosteroids, which are used as a powerful anti-inflammatory drug, but their effect in the lipid profile is unknown. Thus, clinical lipidomics is the utmost approach to evaluate the variation of lipids at a molecular level in several chronic diseases, including AID [91]. The aim of clinical lipidomics is to understand lipid metabolism and mechanisms and identify new putative diagnostic biomarkers and therapeutic targets [92]. Clinical lipidomics is a reliable source of information regarding lipidic alterations and, hopefully, it can be seen as a promising approach to disease diagnostics and therapy monitorization in the near future.

Studies on the variation of RA lipid profile at a molecular level using lipidomics were gathered in this review. English language publications were identified through a computerized search (using PubMed database) until 2020 using the following keywords: lipidomic(s), lipid profile, fatty acid(s), phospholipid(s), sphingolipid(s), ceramide(s), and oxidized, combined with rheumatoid arthritis. Studies that did not report the use of mass spectrometry (MS) techniques were not taken into consideration. To structure this review, the studies were divided in three sections: lipidomic studies on RA serum/plasma samples; lipidomic studies on other types of samples from RA patients; and lipidomic studies on high-risk patients to develop RA.

### 4.1. Lipidomic Analysis of Serum/Plasma in Rheumatoid Arthritis Patients

Serum/plasma is the most important body fluid to portray metabolic changes. The analysis of serum/plasma sheds light on alterations induced by a pathological environment. The majority of the lipidomic studies on RA has been performed in human serum/plasma samples using mainly gas chromatography-mass spectrometry (GC-MS) for FA profiling, comparing RA patients with healthy controls, also including studies that evaluated the effect of FA diet, but not the effect of disease state. Few studies used liquid chromatography-mass spectrometry (LC-MS) for PL profiling, free fatty acids (FFA) and oxidized FA, also comparing data from plasma/serum from RA patients and controls (Table 1).

#### 4.1.1. Fatty Acid Profiling Analysis

The studies that reported the changes in FA profile in RA patients, compared with non-disease subjects, reported similar trends in some FA while opposite trends were reported in other studies. This can also be due to different experimental approaches since some studies investigate the variation of FA in plasma [93,94,95,96], while others reported in phosphatidylcholine (PC) [97] or in total PL [98,99,100,101,102]. The majority of the studies reported a decrease of ω-3 FA, namely a decrease of 18:3 ω-3, 20:5 ω-3 or/and 22:6 ω-3. A decrease of palmitic acid (16:0) was seen in some studies, but was increased in others, while the saturated FA 18:0 was reported to decrease or not change in different studies. The ω-6 FA, such as the arachidonic acid (20:4), showed also contradictory variations. Other FA were also affected by disease, as will be described.

Bruderlein and colleagues investigated the FA profile of serum PL of RA patients [98]. They determined significant decreases of FA 16:0; 18:2 ω-6 and 18:3 ω-3, and significant increases of FA 18:1 ω-9; 20:3 ω-6; 20:3 ω-9 and (20:3 + 20:4) ω-6/18:2 ω-6 ratio [98]. It was suggested that the ω-6 metabolic pathway is altered in RA patients, and it was induced by the persistent inflammation of this disease. The sum of ω-6 FA was constant, thus the disease seemed to stimulate an alteration in the content of different ω-6 FA towards higher unsaturated species, namely 20:3 and 20:4 [98]. When in a cell membrane, 20:3 ω-6 and 20:4 ω-6, have a much higher distortion, conformation wise, than that of 18:2 ω-6, due to the presence of a higher number of unsaturations. Changes in the abundance of these FA may affect membrane properties [103]. Jacobsson and co-workers analyzed the FA composition of serum PC (the major PL class in plasma/serum lipid profile) in RA patients of short and long disease duration [97]. It was verified a significant decrease of FA 16:1; 18:0; 18:2 ω-6; 18:3 ω-3; 20:5 ω-3; 22:5 ω-3; 18:2/20:4 ratio and Σ ω-6 and a significant increase of FA 14:0; 16:0; 18:1 and Σ saturated FA. These changes became more evident with the increase of disease duration [97]. The disturbances in FA of the ω-6 series are in agreement with the previous findings of Bruderlein [98]. The reduced 18:2/20:4 ratio in RA patients was suggested to be related to the degree of inflammation, due to an increase in the FA desaturation/elongation process, which could be the result of higher insulin levels (stimulates desaturation) that have been found in RA patients, or due to an increased synthesis of eicosanoids [104,105]. Suryaprabha and colleagues studied plasma concentrations of essential FA of PL and determined a marked decrease of FA 18:0; 18:3 ω-6; 18:3 ω-3; 20:3 ω-6; 20:5 ω-3 and 22:6 ω-3 [99]. Interleukin-2 dependent T-cell proliferation and tumour necrosis factor and interleukin-2 production can be blocked by PUFA, thus, the reduction of essential FA and their metabolites may contribute to increase the chronic inflammation once T-cell proliferation, tumour necrosis factor and interleukin-2 production may not be inhibited [106]. The low levels of EPA and DHA also contribute to the inflammatory state [99].

Non-esterified FA/FFA profile of serum in RA was also evaluated by using LC-MS lipidomic approaches, and revealed a significant reduction of FA 16:0; 16:1 ω-7; 18:1 ω-9 and 20:4 ω-6 when compared with healthy controls [93]. The FA 20:5 ω-3 and 22:6 ω-3 were reported to have significantly lower concentrations in RA patients as well [93]. These results were associated with SE presence, which suggests that non-esterified FA alterations could be on the onset of RA development.

#### 4.1.2. FA Analysis after a Fasting Period

Changes in FA profile in RA with different diets or in a fasting/non-fasting regimen was also evaluated, since fasting has been shown to have significant beneficial effects in clinical manifestations in RA [107]. Hafström et al. evaluated the serum levels of FA of membrane PL of RA patients after a fasting period [100] and observed markedly reduced levels of FA 20:3 ω-6 and markedly elevated levels of FA 20:4 ω-6 and 20:5 ω-3 comparing with RA patients before fasting and healthy control’s levels [100]. Haugen et al. investigated the possible impact on disease activity of fasting and one-year vegetarian diet in patients with RA [101]. The results showed that, after a vegan diet period, several FA of plasma PL were significantly decreased, such as FA 14:0; 16:0; 16:1; 18:0; 18:1; 18:3 ω-3; 20:1; 20:3 ω-9; 20:3 ω-6; 20:4 ω-6; 20:5 ω-3; 22:0; 22:1 ω-11; 22:4 ω-6; 22:5 ω-6; 22:5 ω-3; 22:6 ω-3; Σ ω-6; Σ ω-3 and Σ saturated FA [101]. A few years forward, Fraser and co-workers evaluated free FA changes in plasma of RA subjects after fasting and assessed the effects upon T lymphocyte proliferation [94]. Long chain ω-3 and ω-6 FA are linked with the inhibition of T lymphocyte function [108,109,110]. However, it is unclear if alterations on total concentration of circulating FFA would influence the immune response. Firstly, Fraser determined marked increases of FFA 14:0; 16:0; 16:1 ω-7; 18:0; 18:1 ω-9; 18:2 ω-6; 18:3 ω-3 and total FFA after a fasting period [94]. Secondly, it was shown that the proliferative response of T lymphocytes was higher with the increase of FA levels. Lastly, in vitro tests revealed that the ratio of unsaturated/saturated FFA had a significant effect upon lymphocyte proliferation. Lymphocyte proliferative responses, after mitogenic stimulation in the presence of (1) only unsaturated FA or (2) only saturated FA, were significantly lower than when stimulated in the presence of a mixture of unsaturated/saturated FA [94]. The practical implications of this finding to in vivo situations remains uncertain.

#### 4.1.3. FA Analysis after Supplementation Intake

The effect of dietary fatty acid was also evaluated in a few studies, and dissimilar variations were found, which may be due to different supplements, methods, and samples. Kremer and co-workers examined the effect of manipulation of dietary FA comparing plasma of RA patients with and without EPA supplementation [95]. As expected, patients taking EPA supplementation had significantly higher levels of plasma EPA than the patients not taking the supplementation [95]. Remans et al. had similar results as obtained by Kremer’s team. Remans compared the FA profile of PL of active RA patients receiving nutrient supplementation containing PUFA, including EPA, and micronutrients, with a group of active RA patients receiving placebo [102]. All patients receiving nutrient supplementation had significant increases of total ω-3 PUFA (20:5; 22:5; 22:6) and decrease of AA [102]. Although there were significant changes in plasma FA, the nutritional supplementation did not improve signs and symptoms of RA patients. On the other side, Jäntti et al. also evaluated the influence of supplementation with evening primrose oil (rich in FA 18:3 ω-6) and determined significantly lower concentrations of serum FA 18:1 and EPA; and markedly increased levels of serum FA 18:2; 18:3; 20:3 and 20:4 [96]. The intake of evening primrose oil had contradictory effects, increasing both anti-inflammatory FA 18:3 and pro-inflammatory AA. The increase of FA 18:3 may be regarded as favorable to help reduce the inflammatory status. However, the increase of AA may be considered as harmful since it is the precursor of pro-inflammatory prostaglandins and leukotrienes [96]. Proudman [111] analyzed the results of an investigation conducted previously by other authors, and therefore did not mention original results to be discussed in this review. PUFA supplementation should have, in theory, beneficial results and improve clinical symptoms because EPA and DHA compete with AA for incorporation in cellular membranes, which leads to a reduction in the synthesis of prostaglandins and leukotrienes, thus reducing the inflammatory state [112]. However, investigators struggle to find the correct dosage of PUFA supplementation proven by different studies (with unequal doses) having different results. Having said that, more studies are needed to ascertain the quantity of PUFA supplementation should be administered to RA patients in order to maximize the results.

#### 4.1.4. Phospholipidomic Profiling Analysis by LC-MS

To our knowledge, there is only one study that reported untargeted lipidomic analysis of plasma PL of RA subjects. Łuczaj et al. [113] determined alterations on the PL profile that reflected a decrease on PC(40:2); PC(40:3) and PC(42:3), and an increase on lyso-PC (LPC) species LPC(16:1); LPC(24:3); PC(34:3); PC(36:3); PC(38:2); PC(38:3); PC(38:4); lyso-phosphatidylethanolamine (LPE) species LPE(16:0); LPE(18:0); phosphatidylethanolamine (PE) specie PE(30:1); phosphatidylinositol (PI) species PI(36:1); PI(36:2); PI(36:3); PI(36:4); PI(38:3); PI(38:4) and sphingomyelin (SM) species SM(d34:2); SM(d38:1); SM(d40:1) and SM(d40:2) [113]. The results suggest a significantly altered PE and PC metabolism with enhanced PL hydrolysis by phospholipase A_2_. In addition, the increase of LPC in RA has already been associated with the induction of cyclooxygenase 2 hence contributing to systemic inflammation [114,115]. PC and PE can also be modified by ROS in oxidative stress conditions [116]. Although the main targets of oxidation are the unsaturated FA chains and PC is more abundant than PE, PE is more reactive over PC since it has a free amino group in the polar head, being a preferable target to suffer modifications induced by oxidative stress conditions and ROS which are enhanced in inflammation. Sphingolipids have pleotropic pro-inflammatory effects. Therefore, the study of its metabolism is very important. Regarding sphingolipids metabolism, other findings worth mentioning are the detection of increased levels of total monohexosylceramides (HexCer, as a sum of HexCer16:0; HexCer16:1; HexCer18:0; HexCer20:0; HexCer22:0; HexCer23:0; HexCer24:0 and HexCer24:1), total ceramides (Cer, as a sum of Cer16:0; Cer18:0; Cer20:0; Cer22:0; Cer23:0; Cer24:0; Cer24:1; Cer25:0 and Cer26:0;) and sphingosine (d18:1) in serum of established RA patients [117].

#### 4.1.5. Identification of Lipid Peroxidation Products by LC-MS

Lipid peroxidation products of AA and linoleic acid in RA patients were evaluated through LC-MS techniques by Charles-Schoeman and co-workers [118]. It was analyzed the presence of hydroxyeicosatetraenoic acids (HETE) and hydroxyoctadecadienoic acids (HODE) in plasma lipoproteins HDL and LDL. The results showed markedly increase levels of oxidation products 5-HETE; 15-HETE; 9-HODE and 13-HODE in both HDL and LDL fractions of RA patients [118]. HETE and HODE contribute to LDL oxidation which, together with their accumulation in HDL particles, may inhibit HDL beneficial function increasing the risk of developing atherosclerotic events [119,120]. Higher concentrations of these lipid peroxidation products in HDL are associated with the decreased antioxidant capacity of such particles. Thus, the raised levels of systemic inflammation, also determined in this investigation, positively correlates with the increased levels of free oxidized FA in HDL and LDL, reassuring the fact that RA is an AID with an exacerbated oxidative environment [118].

Overall, the serum/plasma lipidomic analysis of the studies reported above suggest that RA is characterized by an altered lipid metabolism. Plasma FA alterations were observed and revealed as significant lower levels of FA 20:5 ω-3 and 22:6 ω-3, although some contradictory variations were reported, as for other FA. The plasma PL profile was found to be changed with manifestations on PE and PC metabolism with enhanced PL hydrolysis, in a solely study that used lipidomic approaches.

Some attempts were made regarding the effects of PUFA supplementation to compensate the lower level of ω-3 PUFA in RA, but more studies are needed to understand exactly which dose should be administrated and if, besides FA alterations, there are, in fact, changes concerning disease activity.

Nonetheless, lipid metabolism is undeniably affected in RA. Considering the important role of lipids in inflammation, more studies are needed, particularly using modern lipidomics, which could contribute to unveil the pathophysiology of this disease, find new biomarkers, and also to develop better therapeutic approaches.

**Table 1:** Main lipid variations observed in serum/plasma of RA patients reported in published lipidomic studies, available in PubMed database, using MS approaches.

Reference	Analytical Method	Lipid Extraction Method	N° and Age (Years) of RA Patients	Extra Details of the Study	Molecular Specie	Results	
						↓ Decrease	↑ Increase
FA profiling analysis							
Bruderlein et al. [98]	GC-MS	Gloster and Fletcher method	20 RA Age: 25–66	NSAID treatment.	FA esterified to PL	16:0; 18:2 ω-6; 18:3 ω-3	18:1 ω-9; 20:3 ω-6; 20:3 ω-9; (20:3 + 20:4) ω-6/18:2 ω-6 ratio
Jacobsson et al. [97]	GC-MS	INF	Two groups: 21 RA Age: 25–78 21 RA Age: 3–43	NSAID treatment.	FA esterified to PC	16:1; 18:0; 18:2 ω-6; 18:3 ω-3; 20:5 ω-3; 22:5 ω-3; 18:2/20:4 ratio; Σ ω-6	14:0; 16:0; 18:1; Σ saturated FA
Suryaprabha et al. [99]	GC-MS	Methanol:Chloroform (1:2, *v/v*)	14 RA Mean age: 33 ± 9	Not on drugs when sample collection.	FA esterified to PL	18:0; 18:3 ω-6; 18:3 ω-3; 20:3 ω-6; 20:5 ω-3; 22:6 ω-3	-
Rodríguez-Carrio et al. [93]	LC-MS/MS	MTBE	124 RA Mean age: 52.47 ± 12.76	43 patients with smoking habits.	Plasma FFA	16:0; 16:1 ω-7; 18:1 ω-9; 20:4 ω-6; 20:5 ω-3; 22:6 ω-3	-
Analysis after a fasting period							
Hafström et al. [100]	GC-MS	Methanol:Chloroform (2:1, *v/v*)	14 RA Age: 34–65	No steroids/NSAID treatment for the previous 3 months. 7 days of fasting.	FA esterified to PL	20:3 ω-6	20:4 ω-6; 20:5 ω-3
Haugen et al. [101]	GC-MS	n-butanol (butyl alcohol)	53 RA Mean age: 51	Fasting period of 7–10 days followed by 1-year vegetarian diet.	FA esterified to PL	After vegan diet 14:0; 16:0; 16:1; 18:0; 18:1; 18:3 ω-3; 20:1; 20:3 ω-9; 20:3 ω-6; 20:4 ω-6; 20:5 ω-3; 22:0; 22:1 ω-11; 22:4 ω-6; 22:5 ω-6; 22:5 ω-3; 22:6 ω-3; Σ ω-6; Σ ω-3; Σ saturated FA	-
Fraser et al. [94]	GC-MS	n-hexane	9 RA Age: 31–65	Steroids/NSAID treatment. 7 days of fasting.	Plasma FFA	-	14:0; 16:0; 16:1 ω-7; 18:0; 18:1 ω-9; 18:2 ω-6; 18:3 ω-3; Σ FFA
Analysis after supplementation intake							
Kremer et al. [95]	GC-MS	INF	37 RA Age: INF	NSAID treatment. EPA supplementation.	Plasma FFA	-	With supplementation 20:5
Remans et al. [102]	GC-MS	Bligh & Dyer method	26 RA Mean age: 59.5 ± 11.0	Steroids treatment. Nutritional supplement with PUFA and micronutrients.	FA esterified to PL	With supplementation 20:4	With supplementation Σ ω-3 PUFA (20:5; 22:5; 22:6)
Jäntti et al. [96]	GC-MS	Methanol:Chloroform (1:1, *v/v*)	Two groups: 10 RA Mean age: 50 10 RA Mean age: 38	No NSAID treatment for the previous 7 days. Evening primrose oil/olive oil supplementation.	Plasma FFA	With supplementation 18:1; 20:5	With supplementation 18:2; 18:3; 20:3; 20:4
Phospholipidomic profiling analysis by LC-MS							
Łuczaj et al. [113]	LC-MS	Modified Folch method	9 RA Age: 23–79	No steroids/NSAID treatment. Excluded heavy smokers.	Plasma PL	PC(40:2); PC(40:3); PC(42:3)	LPC(16:1); LPC(24:3); PC(34:3); PC(36:3); PC(38:2); PC(38:3); PC(38:4); LPE(16:0); LPE(18:0); PE(30:1); PI(36:1); PI(36:2); PI(36:3); PI(36:4); PI(38:3); PI(38:4); SM(d34:2); SM(d38:1); SM(d40:1); SM(d40:2)
Identification of lipid peroxidation products by LC-MS							
Charles-Schoeman et al. [118]	LC-MS/MS	Methanol/water	10 RA Mean age: 49.6 ± 11.8	10% of the patients with smoking habits.	Plasma HDL + LDL	-	5-HETE; 15-HETE; 9-HODE; 13-HODE

INF: information not found.

### 4.2. Lipidomic Analysis of Other Types of Samples of Rheumatoid Arthritis Patients

Serum/plasma is undoubtedly the best source of information regarding pathologically induced metabolic alterations. However, the lipidomic analysis of other types of samples (body fluids) and also blood cells offer valuable insights into the disease as well. The works published to date and found in the literature describe changes in lipids, in particular FA, in synovial fluid, platelets, erythrocytes, PBMC, and adipose tissue of human samples (Table 2).

Synovial fluid is extremely important in RA diagnosis. When there is a joint problem, synovial fluid can accumulate, causing stiffness, pain, and inflammation, therefore its analysis should reveal interesting results. Synovial fluid can physiologically change in volume and content which can occur in response to trauma, inflammation, or bacterial/fungal/viral penetrance, leading to an accumulation of selected molecules [121]. Thus, synovial fluid may be considered the body fluid of choice to obtain crucial information. Nevertheless, the collection of synovial fluid (arthrocentesis) is a difficult process for RA patients and even more for healthy volunteers. To the best of our knowledge, no studies reported the comparison of synovial fluids between RA patients and healthy volunteers. There is only one study that used GC-MS to evaluate the FA profile of the synovial fluid comparing it with the FA profile of serum from the same RA patient [122]. Quantitatively (% of total FA esters), the FA composition of the synovial fluid of RA patients was significantly lower, approximately one third of that of serum samples. In both biofluids, the major FA were FA 16:0; 18:1 and 18:2 (the sum of the relative abundances of these FA accounted for nearly 80% of FA composition) while FA 14:0; 16:1; 18:0 and 20:4 were less abundant [122]. The synovial fluid membrane significantly contributes to local lipid content, by releasing lipids to the synovial fluid. Thus, the qualitative composition of synovial fluid and serum of RA is similar [123].

Platelets release pro-inflammatory microparticles after being activated, which in turn interact with leucocytes, promoting joint and systemic inflammation in RA [124]. Hafström and colleagues determined the FA profile of platelets (by GC-MS), after a fasting period and found markedly reduced levels of FA 20:3 ω-6 while AA and 20:5 ω-3 showed the opposite trend, being markedly increased in RA subjects [100]. In this study, the composition of serum and platelets concerning the FA profile was equivalent, suggesting a fast exchange of lipids between platelets membrane and serum.

Erythrocytes are likewise involved in RA pathobiology. Erythrocytes count is positively correlated with joint pathology, as well as with inflammatory biomarkers. There is also an alteration in the lipid distribution within erythrocytes membranes [125]. Masoom-Yasinzai et al. assessed the status of EPA in the membrane of RA erythrocytes and found markedly decreased levels of 20:5 ω-3, as compared with controls, which may suggest an impaired EPA metabolism in these cells [126]. Lee and colleagues investigated if erythrocyte levels of ω-3 PUFA were linked with disease activity and the risk of developing RA [127]. It was found significantly lower concentrations of FA 18:1 ω-9; 18:2 ω-6; 18:3 ω-3; 20:5 ω-3; ω-3 index and 20:5/20:4 ratio, and significantly higher concentrations of FA 14:0; 16:0; 16:1 ω-7; 18:0 and 18:3 ω-6 [127]. The authors determined that the risk of RA was positively correlated with the levels of saturated FA; 14:0; 16:0; 18:0; 16:1 ω-7; 18:3 ω-6; ω-6 PUFA; *trans* 18:2 and *trans* FA and disease activity was positively associated with age [127]. Lastly, Park and co-workers aimed to determine if ω-3 PUFA supplementation improved the clinical outcomes of RA patients [128]. The results showed a significant decrease of FA 18:1 ω-9; 18:2 ω-6; 20: 4 ω-6; total ω-6 PUFA and ω-6/ω-3 ratio, and a significant increase of FA 20:5 ω-3 and total ω-3 PUFA after supplementation [128]. Nevertheless, ω-3 PUFA supplementation did not ameliorate the clinical outcomes, both clinical symptoms and laboratory parameters, of RA patients.

Unlike the previously described investigations, some studies with immune cells, neutrophils [100] and peripheral blood mononuclear cells [94] (PBMC, which include lymphocytes and monocytes), did not reveal statistically significant results, as will be described. Neutrophils are key players in RA pathogenesis, favoring, among others, an oxidative environment and cartilage destruction, as explained before. Hafström and collaborators determined the FA profile of neutrophils after a fasting period. However, the investigation did not report significant results [100]. Changes in the PL composition of the membrane of PBMC directly influence immune cell functions [129] and thus, Fraser et al. evaluated the FA profile of PBMC cellular membranes, but no statistically significant differences in the amount of FA as a percentage of total cellular lipids between RA and healthy subjects were found [94].

Adipose tissue reflects the long-term FA status. Hence, its analysis would indicate if the lipid metabolism alterations derive from the onset of RA, or if they are established later in RA development. Jacobsson and co-workers analyzed the FA composition of adipose tissue of patients with RA which showed a significant decrease of FA 18:2 ω-6; 18:3 ω-3 and total ω-6 [97]. These abnormalities were found to be more severe with the increase of disease duration.

From the results described above, it cannot be concluded if different types of samples show the same metabolic alterations at a lipid level. However, the low levels of FA 18:2 ω-6 and 20:5 ω-3 in erythrocytes are in agreement with the low levels found in adipose tissue and PL serum of RA subjects [97,98]. Furthermore, the increased levels of saturated FA are consistent with previous studies that also found higher levels of these FA in plasma PL of RA [97,130] and lower levels of EPA and other ω-3 FA. Further studies are needed to confirm or not this trend. Changes in PL were not studied at all besides plasma/serum.

**Table 2:** Main lipid variations observed in synovial fluid [122], platelets [100], erythrocytes [126,127,128], neutrophils [100], PBMC [94], and adipose tissue [97] of RA patients reported in published lipidomic studies, available in PubMed database, using MS approaches.

Reference	Analytical Method	Lipid Extraction Method	N° and Age (Years) of RA Patients	Extra Details of the Study	Molecular Specie	Results	
						↓ Decrease	↑ Increase
Kim et al. [122]	GC-MS	Folch method	10 RA Age: INF	-	FFA	-	-
Hafström et al. [100]	GC-MS	Methanol:Chloroform (2:1, *v/v*)	14 RA Age: 34–65	No steroids/NSAID treatment for the previous 3 months. 7 days of fasting.	Membrane FA	Neutrophils: NSD Platelets: 20:3 ω-6	Neutrophils: NSD Platelets: 20:4 ω-6; 20:5 ω-3
Masoom-Yasinzai [126]	GC-MS	Chloroform:Methanol (2:1, *v/v*)	15 RA Age: INF	No steroids/hypolipidemic treatment.	Membrane FA	20:5 ω-3	-
Lee et al. [127]	GC-MS	INF	100 RA Mean age: 48.39 ± 9.69	Steroids/NSAID treatment. 13% of the patients with smoking habits.	Membrane FA	18:1 ω-9; 18:2 ω-6; 18:3 ω-3; 20:5 ω-3; ω-3 index; 20:5/20:4 ratio	14:0; 16:0; 16:1 ω-7; 18:0; 18:3 ω-6
Park et al. [128]	GC-MS	INF	Two groups: 41 RA Mean age: 49.24 ± 10.46 40 RA Mean age: 47.63 ± 8.78	Steroids/NSAID treatment. 15 patients with smoking habits. ω-3 PUFA supplementation.	Membrane FA	With supplementation 18:1 ω-9; 18:2 ω-6; 20: 4 ω-6; Σ ω-6 PUFA; ω-6/ω-3 ratio	With supplementation 20:5 ω-3; Σ ω-3 PUFA
Fraser et al. [94]	GC-MS	Benzene	9 RA Age: 31–65	Steroids/NSAID treatment. 7 days of fasting.	Membrane FA	NSD	NSD
Jacobsson et al. [97]	GC-MS	2% Sulfuric acid in Methanol:Toluene (1:1, *v/v*)	Two groups: 21 RA Age: 25–78 21 RA Age: 3–43	NSAID treatment.	FA	18:2 ω-6; 18:3 ω-3; Σ ω-6	-

NSD: no significant differences; INF: information not found.

### 4.3. Lipidomic Analysis of Erythrocytes from Pre-Clinical Rheumatoid Arthritis Subjects

Accumulating evidence suggests that there is a pre-clinical period of increased levels of RA-related autoantibodies that precedes RA development [131]. This type of antibodies is usually detected 3–5 years prior to RA onset [132,133]. This pre-clinical phase of RA re-enforces the multifactorial aspect of RA pathogenesis by suggesting that genetic and environmental factors act previously to the development of classifiable RA, leading to autoantibodies production [134]. This way, lipidomic investigations in pre-clinical RA subjects, which are considered to have a high risk of developing RA forward in their lives, may reveal promising results that could ultimately lead to preventive interventions (Table 3).

As far as our knowledge goes, there are only three lipidomic studies on pre-clinical RA patients that used GC-MS approaches. Gan and colleagues conducted two different investigations, only one of which obtained reportable results [135,136]. The first study described significantly lower levels of 22:6; 20:5 + 22:6 and total ω-3 FA% in erythrocytes of patients who tested positive for anti-CCP2 autoantibodies (an RA-specific autoantibody) [135]. The results suggest an inverse correlation between ω-3 FA and anti-CCP2 positivity. In the second study, Gan and his team tested the seropositivity for several antibodies and determined that pre-clinical RA patients who were positive for RF and SE showed significantly decreased concentrations of FA 22:5 ω-3; 22:6 ω-3; total ω-3 FA% and 20:5 + 22:6 [136]. Similarly, pre-clinical RA patients who tested positive for anti-CCP2 and SE also had markedly reduced levels of FA 20:5 ω-3; 22:6 ω-3; total ω-3 FA% and 20:5 + 22:6 [136]. Resembling the first investigation, the authors concluded that RF, anti-CCP2 and SE positivity is accompanied by low levels of ω-3 FA, which may be linked with RA pathogenesis and RA-related autoimmunity in the pre-clinical phase of this disease. Recently, Pablo et al. tested the hypothesis that higher levels of long-chain ω-3 PUFA were associated with a lower risk of developing RA [137]. The study found significantly increased levels of FA 20:3 ω-6 and 22:5 ω-3, which were increasing with the approaching of the time of the diagnosis [137]. It was also observed that erythrocyte membrane levels of FA 18:2 ω-6 were inversely correlated with the risk of developing RA, meaning that higher levels of 18:2 ω-6 were associated with a lower risk of RA [137]. The hypothesis proposed by the authors was not confirmed.

In summary, lipidomic analysis on erythrocytes add valuable information to the existing knowledge on the lipid profile of RA and pre-clinical RA. The studies of Gan et al. [135,136] corroborate previous findings in erythrocytes of RA patients who determined low levels of 20:5 ω-3 and ω-3 index [126,127]. However, the investigations of Pablo et al. [137] and Gan et al. [136] present opposing results regarding the FA 22:5 ω-3, revealing the intricacy of the lipid metabolism under both pre-pathological and pathological conditions.

**Table 3:** Main lipid variations observed in erythrocytes from subjects with high risk of developing RA reported in published lipidomic studies, available in PubMed database, using MS approaches.

Reference	Analytical Method	Lipid Extraction Method	N° and Age (Years) of RA Patients	Extra Details of the Study	Results	
					↓ Decrease	↑ Increase
Gan et al. [135]	GC-MS	INF	30 pre-RA Mean age: 45.6 ± 16.5	2 patients with smoking habits.	Anti-CCP2 + 22:6; Σ ω-3 FA%; 20:5 + 22:6	-
Gan et al. [136]	GC-MS	INF	Two groups: 40 pre-RA Mean age: 43.7 ± 15.4 27 pre-RA Mean age: 48.1 ± 13.2	5 patients with smoking habits.	RF + and SE + 22:5 ω-3; 22:6 ω-3; Σ ω-3 FA%; 20:5 + 22:6 Anti-CCP2 + and SE + 20:5 ω-3; 22:6 ω-3; Σ ω-3 FA%; 20:5 + 22:6	-
Pablo et al. [137]	GC-MS	Chloroform:Methanol (2:1, *v/v*)	96 pre-RA Mean age: 51 ± 7.56	29 patients with smoking habits.	-	20:3 ω-6; 22:5 ω-3

NSD: no significant differences; INF: information not found.

## 5. Concluding Remarks and Future Perspectives

By the evidence reported in this review, alteration of the lipid metabolism seems to be a characteristic of RA that is far from being completely elucidated. For some cases, dissimilar lipid changes were reported. Variations between studies may also be related to the experimental approach (whether the data are from the analysis of total FA or only from the esterified fraction) and data analysis process, hence the need for further studies based on standardized protocols. The upcoming studies should be standardized so that the results obtained are more robust and more easily comparable, making possible the identification of reliable biomarkers.

**Figure 2:**
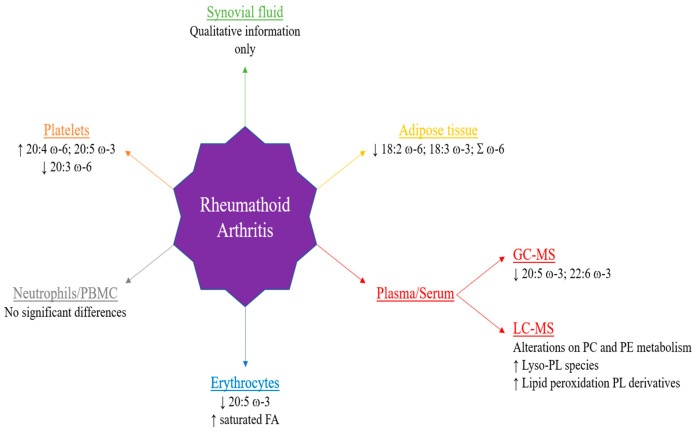
Main lipidomic alterations described in several studies reported in this review. PBMC: peripheral blood mononuclear cells; GC-MS: gas-chromatography mass spectrometry; LC-MS: liquid-chromatography mass spectrometry.

Lipid alterations can be observed even before disease manifestations, which suggests that these molecules are closely linked to the most widespread metabolic changes caused by inflammation (associated with TNF-α) [138,139]. As a result, it is important to study in deep the lipid modulation by RA as it can provide potential markers for early diagnosis of this disease. New biomarker discovery is important once early diagnosis is essential for an early treatment of RA and even for a better prognosis of the disease. All of the reviewed investigations clearly validate the importance of lipids in inflammation, autoimmunity and in the onset (pre-clinical RA) and development of RA. In particular, FA and PE and PC metabolism are primarily affected in this disease, namely a decrease of ω-3 FA, in particular 20:5 ω-3 and 22:6 ω-3 in serum/plasma, increase/decrease of PC and PE species, the increase of oxidized PL and the increase of polar lipids in their lyso form (Figure 2). Additionally, more studies are needed to clarify the effect of lipid supplementation on disease onset and follow-up.

Lipids are important players in inflammation and are modulated in RA. It is very important to continue to advance in this research field: (1) lipidomics of plasma/serum/lipoproteins of patients with RA to characterize the lipidomic signature typical of RA, and in the different stages of the disease to unveil biomarker discovery; (2) lipidomics of immune cells for detailed characterization of changes in their lipid profile to understand how they can correlate with the dysfunction of these cells and the development of RA. In this way, clinical lipidomics, by allowing a detailed lipidome profiling of RA, could be a great contributor and add value on the personalized medical field, leading to a more precise and early diagnosis, management of disease progression, and evaluation of therapy strategies and outcomes.

## Data Availability

Not applicable.
